# Interprofessional Pharmacokinetics Simulation: Pharmacy and Nursing Students’ Perceptions

**DOI:** 10.3390/pharmacy6030070

**Published:** 2018-07-20

**Authors:** Cheryl D. Cropp, Jennifer Beall, Ellen Buckner, Frankie Wallis, Amanda Barron

**Affiliations:** 1 McWhorter School of Pharmacy, Samford University, 800 Lakeshore Drive, Birmingham, AL 35229, USA; ccropp@samford.edu; 2 Ida Moffett School of Nursing, Samford University, 800 Lakeshore Drive, Birmingham, AL 35229, USA; ebuckne2@samford.edu (E.B.); abarron@samford.edu (A.B.); 3 University of Alabama at Birmingham Hospital, NP1333, 1802 6th Avenue South, Birmingham, AL 35249-7010, USA; fwallis@uabmc.edu

**Keywords:** interprofessional education, pharmacy education, nursing education, pharmacokinetics, simulation

## Abstract

Interprofessional practice between pharmacists and nurses can involve pharmacokinetic dosing of medications in a hospital setting. This study describes student perceptions of an interprofessional collaboration pharmacokinetics simulation on the Interprofessional Education Collaborative (IPEC) 2016 Core Competencies. The investigators developed a simulation activity for senior undergraduate nursing and second-year pharmacy students. Nursing and pharmacy students (*n* = 54, 91 respectively) participated in the simulation using medium-fidelity manikins. Each case represented a pharmacokinetic dosing consult (vancomycin, tobramycin, phenytoin, theophylline, or lidocaine). Nursing students completed head-to-toe assessment and pharmacy students gathered necessary information and calculated empiric and adjusted doses. Students communicated using SBAR (Situation, Background, Assessment, and Recommendation). Students participated in debrief sessions and completed an IRB-approved online survey. Themes from survey responses revealed meaningful perceptions in all IPEC competencies as well as themes of safety, advocacy, appreciation, and areas for improvement. Students reported learning effectively from the simulation experience. Few studies relate to this type of interprofessional education experience and this study begins to explore student perceptions of interprofessional education (IPE) in a health sciences clinical context through simulation. This real-world application of nursing and pharmacy interprofessional collaboration can positively affect patient-centered outcomes and safety.

## 1. Introduction

Interprofessional education (IPE) is defined by the World Health Organization as “when students from two or more professions learn about, from, and with each other to enable effective collaboration and improve health outcomes” [1]. IPE is an important aspect in current health education and is necessary to train our students for the collaborative practice environment.

The simulation environment provides a unique opportunity for students to apply information they have learned in a traditional lecture-format environment to the setting of a patient care scenario. The 2016 Interprofessional Education Collaborative (IPEC) Core Competencies for Interprofessional Collaborative Practice serve as a guidance document for developing IPE activities [2]. 

### 1.1. Interprofessional Education at Samford University College of Health Sciences

**Figure 1:**
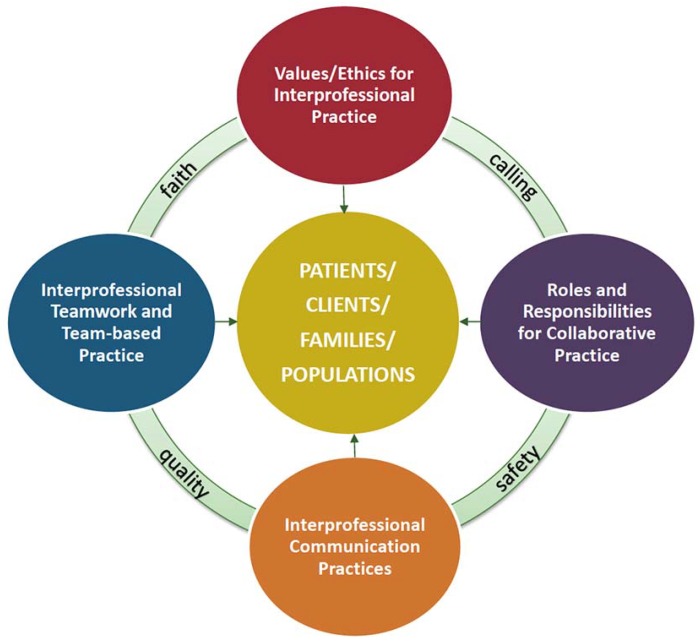
Samford University College of Health Sciences Model of Interprofessional Education.

In 2016, the College of Health Sciences (CHS) at Samford University moved to a shared facility. Since the move, the four schools (Pharmacy, Nursing, Health Professions, and Public Health) have developed several IPE experiences that include discussions and simulations. Simulations such as the one described here take place in a multifunction lab of a shared CHS simulation center. The 22,000-square-foot Experiential Learning and Simulation Center offers discipline-specific and interdisciplinary learning opportunities. Students engage at a variety of levels—from lab-based learning with low fidelity models, to simulated patient interactions, to complex high-fidelity simulations. The CHS Interprofessional workgroup was formed in 2013 and has developed a model for IPE at the CHS. We believe that our faith and calling inform and connect our work. We also incorporate quality and safety since quality improvement and patient/client/population safety are crucial, pervasive aspects of providing care. CHS graduates apply relationship-building values and the principles of team dynamics to perform effectively in different team roles. The CHS has developed a model for IPE below (Figure 1).

In 2018, CHS colleagues proposed a staged model of IPE in which students grow in competencies across longitudinal experiences and maturing practice [3]. These stages are (a) Discuss—discuss the varied roles of IPE participants; (b) Collaborate—working together to analyze a case study and make recommendations; (c) Simulate—create a live action scenario with interprofessional team members and (d) Practice—integrate IP roles in practice settings in preceptorship and during transition to care roles. The experiences proposed here build additional strength in the third and fourth stages of Simulate and Practice transition.

### 1.2. Interprofessional Simulation in Literature

Simulation activities can create an opportunity for disciplines to interact and learn together prior to the completion of their degree training [4]. Smithburger and colleagues developed an elective interprofessional experience with teams that included nursing and pharmacy students. The teams were tasked with managing a complex patient case assuming their respective professional roles. Students were surveyed on their acceptance and impressions of the use of the simulation; they agreed or strongly agreed that the simulation improved their interprofessional communication and confidence in caring for a patient in a team.

In a study of nurse–physician relationships simulation was found to transform interprofessional attitudes [5]. The simulation experiences were designed to encourage open communication, shared information, and collaborative decision-making. Trust developed through the experience and reported attitudes were more positive. This study further investigated stereotype changes which was beyond the scope of the current work. In another study, five best practice components were used to structure an IPE simulation. High quality designs included debriefing practices, interprofessional education applications, validation of outcome measures, student satisfaction, and long-term information retention [6]. The current study utilizes best practices and presents initial student perceptions toward building effective IPE programs.

### 1.3. Nursing and Pharmacy Collaborations in Literature

Reports of effectiveness and perceptions of IPE are becoming increasingly available in the literature. Numerous articles report positive change in student attitudes, breadth of understanding, and the development of trust through interprofessional collaborative educational experiences [7]. Authors reported significant improvement in student attitudes of cultural competence, understanding of roles, interprofessional communication and teamwork.

Kostoff and colleagues incorporated SBAR (Situation, Background, Assessment, Recommendation) into their simulation to determine its impact on self-perception by pharmacy students of interprofessional competence and their reactions toward interprofessional collaboration [8]. Nursing and pharmacy students used the SBAR communication tool to share information to develop a patient care plan. Pharmacy student respondents reported that they felt it was a valuable experience, and that they planned to use SBAR in their future role as a pharmacist.

Interprofessional education simulation has shown benefits to a broad representation of disciplines and content focus. Thurston and colleagues reported specific professional stereotypes reported by nursing and pharmacy students who were naïve to IPE experiences [9]. They utilized a Student Stereotypes Rating Questionnaire and although the results were generally positive, there were significant differences noted by each profession. In a joint nursing and pharmacy IPEC job-shadowing practice experience, results showed effectiveness in increasing IPEC competencies [10]. Poirier and colleagues found differences in student self-assessment and faculty assessment of performance in a nursing–pharmacy interprofessional error disclosure in a simulation training program with recommendations to continue [11]. Both reports recommended increased IPE opportunities to include evaluative strategies. 

Wilson and colleagues reported significant improvement in medication safety through an interprofessional collaboration between nurses and pharmacists in practice [12]. These authors conducted focus groups, and found that “knowing about and valuing the skills and responsibilities of other team members and respecting each person’s unique contribution to the work of the team can lead to more effective communication and collaboration in the context of medication safety.” Bell and colleagues described collaboration through interprofessional medication reviews in primary care [13]. In a recent report, authors described a similar positive effect for hospital-based simulation for nursing and pharmacy students [14].

Meyer and colleagues assessed the impact of a simulation with nursing and pharmacy students on their perceptions of interprofessionalism as well as the impact on knowledge of pharmacology [15]. There was a statistically significant increase in interprofessionalism, as determined by mean Attitudes Toward Health Care Teams score post-simulation compared to pre-simulation. Perceptions of the simulation were overall favorable, and over 90% of students reported that the simulation increased their knowledge of pharmacology.

Zuna and Holt investigated the use of a pharmacokinetic simulator to demonstrate one- and two-compartment drug behavior to undergraduate pharmacology students [16]. The students had received instruction in pharmacokinetic principles and calculations in a previous course. Students completed a self-assessment of their confidence and competence in working with pharmacokinetic concepts and calculations. The students self-reported a significant increase after the simulation in their competence in the math skills of pharmacokinetics, and their perceived understanding of the material compared to their perceptions before the simulation.

There have been no studies identified that investigate collaboration between nursing and pharmacy students on the topic of pharmacokinetic dosing in a simulated educational setting. Therefore, our research of this topic is groundbreaking and provides guidance on an important, useful educational activity in an effort to provide safe and effective treatment to patients.

This study describes student perceptions of an interprofessional pharmacokinetics simulation. Student perceptions of the session content, interprofessional collaboration, and the use of simulation were sought.

## 2. Materials and Methods

The investigators developed a simulation activity for senior undergraduate nursing and second-year pharmacy students. This was the second year for this activity. Objectives for the pharmacy students were to: (1) provide optimal drug dosing using population and patient-specific pharmacokinetic parameters; and (2) communicate effectively in the care of a patient. Objectives for nursing students were to: (3) apply principles of leadership through interprofessional collaboration, and (4) promote medication safety through interprofessional collaboration with pharmacy in patient-centered care.

### 2.1. Development of the Simulation and Objectives 

In preparation for the simulation, we hypothesized that pharmacy and nursing students would demonstrate increased interprofessional collaboration in all the IPEC Core Competencies. We further anticipated that the competency of interprofessional communication would increase significantly with addition of shared content and best practices in medication administration and pharmacokinetic dosing. The first offering was in Spring 2017 and students’ anecdotal reports were highly positive. In particular the debrief sessions included all students sharing their perceptions of the collaboration, demonstrating the collegiality that had developed among them. Based on this pilot experience, faculty chose to continue the offering and add formal qualitative measure to evaluate the simulated IPE experience.

In Spring 2018, the activity was offered to 54 nursing and 91 pharmacy students over the course of three consecutive days. The simulation took place in a five-bed flex lab using medium-fidelity manikins. Each manikin represented a different case for which the pharmacy student had been consulted to initiate or adjust pharmacokinetic dosing. The drugs for which doses were to be calculated were vancomycin, tobramycin, phenytoin, theophylline, and lidocaine. The lidocaine case was developed for the Spring 2018 activity; in Spring 2017, the fifth case was a patient receiving vancomycin and gentamicin. The cases were developed in conjunction with the instructor of record for the pharmacokinetics course, who also co-led the lab activity. Each case was designed to reinforce a pharmacokinetic principle or to provide an opportunity to practice completing a pharmacokinetic consult as would take place in an inpatient setting. Each case also had embedded in it an error or omission to provide an aspect of medication safety.

### 2.2. Case Studies and Core Competencies

The simulation activity was created for senior undergraduate nursing and second-year pharmacy students and was congruent with curriculum objectives for each. Nursing students had completed numerous clinical experiences and were able to integrate care expectations in the collaboration. Pharmacy students applied content on pharmacokinetics and collaborated by providing current state-of-the-science evidence-based information on nursing students’ questions about medication parameters. For example, both groups were concerned with monitoring for adverse reactions or side effects and both reported they learned specific new information through the questions and answers on specific drugs. This activity takes place as part of a nursing leadership and management course, and an integrated pharmacy applications lab course. Pharmacy students completed a pharmacokinetics course the semester immediately preceding this simulation. Nursing and pharmacy students were assigned to certain days so each participated only once in the simulation. Nursing students began a head-to-toe assessment of the patient. Pharmacy students began the simulation in a classroom where they received instructions, group assignments and a worksheet to be completed. The worksheet contained a brief case vignette and space on which they could document information collected from the nursing students and the chart, as well as space for calculations. The case vignette included the pharmacokinetic consult with medical diagnosis, physical parameters, and laboratory findings. Five case studies were developed collaboratively by the pharmacy–nursing faculty team.

**Case 1: Vancomycin**

The patient is a 64-year old male who was hospitalized 3 days ago while on vacation in Florida for an infection in a wound on his leg. Further evaluation revealed MRSA in the wound. In addition to home meds for hypertension and ischemic heart disease, patient was placed on vancomycin with peak and trough targets.

**Case 2: Theophylline**

The patient is a 22-year-old female who was admitted to your hospital for an asthma exacerbation. She reports to you that she has not had her prescriptions refilled and ran out of her medications 1 week ago. Pharmacy consult included assessing adherence/compliance, determining dosage for bolus and maintenance theophylline, and discussion with patient about plans for discharge and reducing exacerbations. 

**Case 3: Tobramycin**

The patient is a 24-year old female who resides in a long-term care facility secondary to quadriplegia. She was hospitalized for a urinary tract infection and the urine culture grew *Pseudomonas aeruginosa*. Home meds included oxybutynin, docusate sodium, and, baclofen with pharmacy to dose tobramycin.

**Case 4: Phenytoin**

The patient is a 30-year-old male who is admitted for a closed head trauma and has developed generalized tonic-clonic seizures. He was initially placed on lamotrigine but has not responded well to that. He was started on phenytoin a couple of days ago, but his seizure frequency increased on the second day of therapy. There are lab profiles and a consult for pharmacy to dose phenytoin.

**Case 5: Vancomycin + gentamicin (Spring 2017)**

The patient is a 10-year-old female admitted for osteomyelitis. Consult for pharmacy to dose vancomycin and gentamicin. Pharmacy and nursing collaboration with family to plan for discharge and monitor long-term side effects.

**Case 6: Lidocaine (Spring 2018)**

The patient is a 70-year-old female who resides at a local long-term care facility and is admitted for ventricular tachycardia. Consult for pharmacy to dose lidocaine to a steady-state concentration of 2 mg/L. The patient also had suspected *C. difficile* infection.

Pharmacy students then entered the simulated healthcare environment and communicated with nurses on duty. Each patient case has a team comprised of nursing and pharmacy students. They gathered the necessary information on patient status from the nursing students as well as the chart, and then conferred to calculate empiric or adjusted doses of the aforementioned medications for the patient. Once the dose was calculated, the pharmacy students communicated the planned new dosing regimen (including administration and monitoring parameters) to the nursing students using SBAR. Necessary collaborations with additional health professionals (MD, NP, or PA) were noted. Nursing and pharmacy students both participated in the debrief session including collaborative “rounds” where each discipline identified their top priority problems related to the patient case identified during the simulation. They discussed roles and responsibilities, patient-centered values, communication processes, and criticality of teamwork. Complexity in the simulation included error detection, verification of patient data, appreciation of changing status, and dosage parameters associated with age, body weight, and organ function. Both the simulation and debrief session were facilitated by nursing and pharmacy faculty, providing assistance as needed on content and the collaborative process. In the debrief, any within-profession jargon was addressed and clarified. Lack of actual patients and the absence of a physician in the consultation limited communication, however, and form the basis for our future plans.

In addition to basic case components, several cases had embedded errors or risk that students were expected to identify. For example, nursing students noted to pharmacy that potassium was still being administered even though potassium levels had reached normal range. In another example a mistake was detected when Zosyn (piperacillin/tazobactam) was substituted for Zofran (ondansetron) in the medications available for administration, even though the patient had a penicillin allergy. These safety “near misses” further strengthened the values, teamwork, and communication needed to correct medication errors by both nursing and pharmacy professions.

### 2.3. Surveys of Student Perceptions

A link to a 10-item survey was emailed to students (pharmacy) or available via the course learning management system (nursing). The survey was administered via SurveyMonkey. At the end of each lab session, the students were asked to complete the survey and given time in class to do so. The following questions were included in the survey (Table 1):

The study was conducted in accordance with the Declaration of Helsinki, and the University’s Institutional Review Board approved this study as exempt since student responses were collected anonymously with no identifying information. The investigators individually gathered the results and identified themes among the responses. Student feedback was analyzed using content analysis for purposes of improving the teaching-learning environment and enhancing our knowledge of IPE and IPEC competencies. Results of the content analysis are presented below.

### 2.4. Significance of the Project

The safety and quality of patient care is dependent upon shared communication and understanding between health care professionals. Our project served as a template for the incorporation of interprofessional education standards into a new curriculum for the McWhorter School of Pharmacy, currently under development and scheduled for implementation in the fall of 2019. Additionally, Samford University’s nursing program is exploring curriculum innovations and revisions, especially targeting opportunities for interprofessional experiences. The simulation served as a model for nursing and pharmacy interactions between each other and other health professions. This project represents a unique type of interprofessional simulation between pharmacy and nursing students related to pharmacokinetics. Interprofessional education plays an important role in both curricula, and is necessary to meet the standards for pharmacy school accreditation. Since both schools at our institution are exploring curricular revisions, this project can serve as a model for other simulations in the future. 

## 3. Results

### 3.1. Sample and Response Rate

One hundred nineteen out of 145 students participated in the survey, giving a response rate of 82% (Spring 2018). Seventy-seven of the respondents (64.7%) were pharmacy students while 42 respondents (35.3%) were nursing students. Forty-two of the 54 (77.8%) nursing students and 77 of the 91 (84.6%) pharmacy students who participated in the simulation completed the survey.

### 3.2. Themes from Survey Responses

Themes are listed in Table A1 and Table A2.

Five major areas emerged from the overall thematic analysis for the research: Interprofessional collaboration; interprofessional communication; values and ethics; roles and responsibilities; and teams and teamwork. Each of these areas relates to the Samford University College of Health Sciences IPE framework and core competencies. Other themes that arose from the data included: Safety; advocacy; appreciation for colleagues; patient-centered care; knowledge for practice; applying knowledge and evidence; professionalism; and improvements for the future. Each sample quote was selected based on a consensus of discussion of relevant themes that emerged. The data were not assessed quantitatively, therefore, frequency counts were not completed.

Interprofessional collaboration emerged as the overarching theme from the IPE event. Both pharmacy and nursing students found a common value in the collaboration efforts between the professions. They expressed the importance of working together to provide the best outcomes for the patient. The students also voiced the importance of combining discipline-specific knowledge to increase the level of care for patients.

*“I learned that interprofessional collaboration can maximize the care at higher level versus working as two different units.”*

(Pharmacy student)

*“Interprofessional collaboration is vital to the health of our patients, as certain professions have more in-depth knowledge and different scopes of practice than others.”*

(Nursing Student)

Participants also expressed an appreciation for interprofessional communication between the groups of students. The students recognized the importance of communicating with each other concerning patient status to ensure safe treatment for the patients.

*“I learned the importance of communicating with nursing and other staff about monitoring labs and administering doses.”*

(Pharmacy student)

*“Pharmacy and nursing must have good communication between each other to ensure best possible care of the patient.”*

(Nursing student)

Several students voiced interest in the different values and ethical concerns that were prioritized in the opposite profession.

*“I recognized the difference between what nurses prioritize and what pharmacists generally prioritize.”*

(Pharmacy student)

*“I learned a lot about drug contraindications that I had never given thought before ... and when seeing patient allergies as well as medications ordered that a patient should not be receiving due to another medication ordered.”*

(Nursing student)

Roles and responsibilities of both professions also emerged from the analysis. Students expressed strengths of their own profession, while also pointing out the strengths of the opposite profession throughout the scenario.

*“We were the medication experts. Our strengths included correct dosing and side effects.”*

(Pharmacy student)

*“Our strengths were in assessment and specifics of care.”*

(Nursing student)

*“Nursing and Pharmacy work well together to identify patient problems. Nursing seemed to focus on the physical status of the patient while pharmacy tended to focus on the patients values. Together it made for a nice blend of patient centered care.”*

(Pharmacy student)

*“I learned that pharmacy is a good resource when you have questions about drugs and that they are very knowledgeable about the medications we were giving.”*

(Nursing student)

*“I loved to see how the pharmacy students truly focused on the medication and pharmacokinetics but I also loved showing them how the patient as a human being matters.”*

(Nursing student)

The final major area that emerged during content analysis of the data was teams and teamwork. Participants expressed the importance of working as a team to improve patient outcomes. The students recognized the need for specialized skills set, but also the need to put the individual skills together to create a joint treatment plan.

*“I learned about the complex and rewarding relationship of the nursing/pharmacy team and how our input can successfully treat a patient.”*

(Pharmacy student)

*“It really does take a team and good communication skills to improve a patient’s treatment plan.”*

(Nursing student)

In addition to the major areas that emerged during data analysis, a number of other themes were noted. Students from both professions noted an increase in knowledge for practice and expressed strength in applying knowledge and evidence from the IPE event.

*“This was an excellent kinetics refresher.”*

(Pharmacy student)

*“It increased my knowledge of pharmacology, dosing, interactions, and complications of therapy.”*

(Nursing student)

*“Strength was using knowledge and applying to case studies.”*

(Pharmacy student)

*“I learned more about what pharmacy does and how much of a resource they are to our nursing practice in medication administration.”*

(Nursing student)

Professionalism and appreciation for colleagues also emerged as themes from the IPE simulation event. Students conveyed the importance of holistically caring for individuals and using their specific skills to create the best patient outcomes. Participants also recognized the value other professions provide to the overall well-being of the patient. There was also acknowledgment of the other profession creating a different perspective of patient care.

*“I need to do a better job at looking at the patient as a whole instead of basing it off of the limited numbers that I see.”*

(Pharmacy student)

*“It’s important that we’re knowledgeable about what other professions know and are capable of doing to maximize the outcomes of our patients.”*

(Nursing student)

*“I learned that all the different health professionals have something different to offer and it is so helpful when we put our ideas together.”*

(Pharmacy student)

*“Being able to be around pharmacy was nice concerning discussion of patient because they bring a new perspective.”*

(Nursing student)

Themes of safety, advocacy and patient-centered care also emerged as themes from the data analysis. Participants expressed an awareness of interprofessional teams’ role in the safe and effective care of patients.

*“In order to make proper clinical decisions, it is important to verify the complete patient information with the nursing staff.”*

(Pharmacy student)

*“Effective communication is integral to good and safe care.”*

(Nursing student)

*“You need to treat the patient not necessarily the numbers you see. Interprofessionalism is also very important in treating the patient because the other members can tell you information that is not necessarily on the chart.”*

(Pharmacy student)

*“I learned how to properly voice patient situation and report to professionals from a different discipline.”*

(Nursing student)

*“Nursing and Pharmacy work well together to identify patient problems. Nursing seemed to focus on the physical status of the patient while pharmacy tended to focus on the patients’ [lab] values. Together it made for a nice blend of patient centered care.”*

(Pharmacy student)

Students were asked to explain what improvements could be made on the individual level and also for the simulation experience as a whole. Students expressed the need for greater communication with team members in the future, as well as increasing confidence in interacting with the other profession. In addition, both pharmacy students and nursing students conveyed the need to improve the use of the SBAR communication technique when reporting on the patient. Finally, students of both profession recognized the need for the nursing students to play a larger role in the simulation experience.

*“Truly being engaged when interacting with the nursing students and members of other health professions. Not feeling uncomfortable with things I am unfamiliar with.”*

(Pharmacy student)

*“I can improve in my confidence concerning discussing patient findings with pharmacy. I want to always have it all together and there were some questions that my team did not have the answers to and that put down my confidence slightly.”*

(Nursing student)

*“Make sure that the nursing students know that what they are doing matters. Give them a bigger role in the simulation.”*

(Pharmacy student)

*“I feel like a little more planning on how to optimize the use of the nurses in the simulation would be very beneficial to the simulation experience for both professions.”*

(Nursing student)

Participants were also asked to explain what areas of the simulation experience was preferred over a traditional lecture. Many students stated that they enjoyed the simulation experience as it allowed hands-on practice with real-life scenarios to encourage collaboration and critical thinking.

*“Simulation give insight on real world situations that I see in work quite often. It is beneficial to see that we are being prepared based on real cases.”*

(Pharmacy student)

*“I learn well with hands on activities so simulations help me apply what I’ve read or learned in class to what I will actually be doing.”*

(Nursing student)

*“I like how it is hands-on application and gives me the ability to work with other people.”*

(Pharmacy student)

*“I like hands on learning and doing things physically helps me feel more comfortable in hospital situations because it gives me more practice.”*

(Nursing student)

Although most students voiced preference for learning in the simulation environment, some students expressed the need for classroom lectures to set the foundational knowledge.

*“Lecture sessions set the foundation for us to understand the material.”*

(Pharmacy student)

*“It gives us the appropriate information to perform well in simulation.”*

(Nursing student)

## 4. Discussion

No studies were identified that investigated this collaboration between nursing and pharmacy students on the topic of pharmacokinetic dosing in a simulated educational setting, however there are numerous reports of interprofessional simulations on other topics between pharmacy and nursing students.

The five major themes from the data correlate with the CHS IPE framework. Other themes arose as well, which were investigated in other studies and allow for comparison. In the interest of highlighting one of the IPEC competencies most prominent in this study, it would likely be communication. This was one of the objectives for the pharmacy students (“communicate effectively in the care of a patient”) and was an overarching theme in the students’ comments. Pharmacy students had been taught the SBAR tool prior to the simulation to provide a common technique used by the nursing students. Communication was also the necessary vehicle for certain aspects of medication safety, such as the patient who was receiving potassium despite lab results indicating it was in the desired range.

This current simulation meets several of the IPEC sub-competencies for communication, such as:

- Choose effective communication tools and techniques, including information systems and communication technologies, to facilitate discussions and interactions that enhance team function.
- Communicate information with patients, families, community members, and health team members in a form that is understandable, avoiding discipline-specific terminology when possible.
- Express one’s knowledge and opinions to team members involved in patient care and population health improvement with confidence, clarity, and respect, working to ensure common understanding of information, treatment, care decisions, and population health programs and policies.

Thurston and colleagues explored specific professional stereotypes reported by IPE-naive nursing and pharmacy students [9]. Pharmacy students viewed pharmacists significantly more favorably than nursing students viewed pharmacists in each area except for ability to work independently. In contrast, nursing students viewed nurses significantly less favorably than pharmacy students viewed nurses in academic ability and practical skills. The current study did not reveal more or less favorable perception of one discipline’s skills or abilities. Instead students recognized their own strengths while also recognizing those of the other discipline.

Meyer and colleagues reported an overall favorable response to their interprofessional simulation to increase knowledge of pharmacology [15]. The current study reported a similar favorable response in knowledge for practice and in applying knowledge/evidence.

Effective communication was one of the objectives of this current simulation. Kostoff and colleagues reported that pharmacy students gained confidence in using SBAR and planned to incorporate it into their practice as future pharmacists [8]. The current study provided similar results. Not only did pharmacy students agree that the simulation improved their understanding of the SBAR tool, but they responded that they plan to use SBAR as a future pharmacist in healthcare communication. Nursing students had prior exposure to the SBAR format for communicating intra-professionally in patient hand-off but had not used it for interprofessional collaboration. One of the strengths of SBAR is its application to the patient’s status. The giver must use critical thinking to determine the priority situation, assessment, and recommendation. The giver must select information for patient status to effectively communicate the immediate patient care issue. For nursing students it meant distilling their assessment to a focused communication to the pharmacists reflecting a medication concern. For the pharmacists their communications were strengthened by the common format and recommendations were readily understood in the context of the patient’s overall care. Both groups agreed they needed more practice in use of SBAR.

### Implications for Interprofessional Education

Nursing has welcomed interprofessional collaboration and IPE precisely because of its function on the frontline of patient-centered care. IPE experiences at the senior level can build on interprofessional communication approaches such as SBAR format. Nursing students at the senior level are integrating professional and clinical competencies and are well preparing to collaborate with interprofessional colleagues. In this study student perceptions specifically noted increased appreciation for their colleagues and their knowledge base in pharmacology, thus building trust and laying the groundwork for effective collaborative practice with pharmacists in the future. 

Pharmacy has also welcomed interprofessional collaboration and education well in advance of its requirements in accreditation standards for schools of pharmacy. Recognizing the interactions that advanced practice pharmacy students and practicing pharmacists have with other health care providers, it is in the best interest of these students to provide them with opportunities to practice those earlier in the curriculum. The topic of pharmacokinetics provides an excellent setting for interprofessional education as this is a task where pharmacy and nursing professionals routinely interact.

Together, pharmacy and nursing students gained a higher appreciation for the others in their shared values of patient centered care, in their complementary roles, in general and applied knowledge of specific patients’ needs, and in the teamwork required for effective and safe, high quality care. As educators, it is incumbent upon us to emphasize the professionalism of collaboration and importance of clear communication of within-profession jargon to the other profession in the development of collaborative IPE experiences. This simulation has provided preliminary support for further development of pharmacy–nursing simulations and collaborations in pharmacokinetics.

Future plans include incorporating standardized patient and a standardized physician provider to enhance real-world communication across all roles of medication prescribing, consulting, dispensing, and administration. Future plans also include developing evaluative measures to capture actual growth in collaborative skills and document improvements in patient safety outcomes.

## Figures and Tables

**Table 1:** Survey questions.

1.	What is your major field of study (nursing/pharmacy)?
2.	What did you learn today?
3.	What were your strengths during this activity?
4.	What were your areas for improvement during this activity?
5.	What recommendations for changing this activity do you have?
6.	What did you learn about interprofessional collaboration?
7.	What did you learn about the clinical application of kinetics in this simulation?
8.	What is the one thing you like better about lecture sessions as compared to simulation sessions?
9.	What is the one thing you like better about simulation sessions as compared to lecture sessions?
10.	Do you have any additional comments about the simulation or suggestions for improving the interprofessional education in the College of Health Sciences?

## Appendix A

**Table A1:** Themes and pharmacy/nursing quotes on IPEC competencies and concepts.

Theme	Pharmacy	Nursing
Interprofessional collaboration	“We learned what information we needed from the nurses and what information nurses needed from us.”	“Interprofessional collaboration is vital to the health of our patients, as certain professions have more in-depth knowledge and different scopes of practice than others.”
	“I learned that interprofessional collaboration can maximize the care at higher level versus working as two different units.”	
Interprofessional communication	“I learned the importance of communicating with nursing and other staff about monitoring labs and administering doses.”	“Effective communication is integral to good and safe care.”
		“Pharmacy and nursing must have good communication between each other to ensure best possible care of the patient.”
Values/Ethics	“I recognized the difference between what nurses prioritize and what pharmacists generally prioritize.”	“I learned a lot about drug contraindications that I had never given thought before...and when seeing patient allergies as well as medications ordered that a patient should not be receiving due to another medication ordered.”
Roles/Responsibilities	“I learned about the nurse’s thought processes and how we can work together for best care of the patient.”	“Our strengths were in assessment and specifics of care.”
	“Importance of communication with other professions and the differences in what other professions consider pertinent information versus what we as pharmacists think is the most important information.”	“I loved to see how the pharmacy students truly focused on the medication and pharmacokinetics but I also loved showing them how the patient as a human being matters.”
	“Nursing seemed to focus on the physical status of the patient while pharmacy tended to focus on the patients values. Together it made for a nice blend of patient centered care.”	“I learned that pharmacy is a good resource when you have questions about drugs and that they are very knowledgeable about the medications we were giving.”
	“Nursing and Pharmacy work well together to identify patient problems.”	
	“We were the medication experts. Our strengths included correct dosing and side effects.”	
Teams/Teamwork	“It takes a team to treat a patient.”	“I learned how pharmacy can be consulted for med errors and medication alterations in the clinical setting.”
	“I learned how to effectively work in a team with not only other pharmacy students, but also inter-professionally with the nursing students.”	“It really does take a team and good communication skills to improve a patient’s treatment plan.”
	“I learned about the complex and rewarding relationship of the nursing/pharmacy team and how our input can successfully treat a patient.”	
	“I have learned that collaborative team work is essential in developing an optimal patient care plan.”	
	“Teamwork makes the dream work. Everyone’s has a role to play and should be appreciated.”	
Knowledge for practice	“This was an excellent kinetics refresher.”	“It increased my knowledge of pharmacology, dosing, interactions, and complications of therapy.”
Applying knowledge/evidence	“Strength was using knowledge and applying to case studies.”	“I learned more about what pharmacy does and how much of a resource they are to our nursing practice in medication administration.”
		“Being able to be around pharmacy was nice concerning discussion of patient because they bring a new perspective.”
Professionalism	“I need to do a better job at looking at the patient as a whole instead of basing it off of the limited numbers that I see.”	“It’s important that we’re knowledgeable about what other professions know and are capable of doing to maximize the outcomes of our patients.”
	“I learned that it is very very important and each profession has a place to make the entire process work.”	“I loved to see how the pharmacy students truly focused on the medication and pharmacokinetics but I also loved showing them how the patient as a human being matters.”
Appreciation for colleagues	“I learned that all the different health professionals have something different to offer and it is so helpful when we put our ideas together.”	“I learned more about what pharmacy does and how much of a resource they are to our nursing practice in med administration.”
	“The patient always needs to be treated and not just the numbers.”	“The patient always needs to be treated and not just the numbers.”
		“Learned how to properly voice patient situation and report to professionals from a different discipline.”
Patient-centered care	“Nursing and Pharmacy work well together to identify patient problems. Nursing seemed to focus on the physical status of the patient while pharmacy tended to focus on the patients’ [lab] values. Together it made for a nice blend of patient centered care.”	“Our strengths were in assessment and specifics of care.”
	“I learned about a nurse’s thought process when It comes to assessing a patient and how we can work together to get the best treatment for a patient.”	“I learned about the importance I communicating with pharmacy to ensure our patient receive the best care.”
Safety	“In order to make proper clinical decisions, it is important to verify the complete patient information with the nursing staff.”	“Effective communication is integral to good and safe care.”
Advocacy	“You need to treat the patient not necessarily the numbers you see. Interprofessionalism is also very important in treating the patient because the other members can tell you information that is not necessarily on the chart.”	“We learned how to communicate and how to have a voice.”
	“The patient always needs to be treated and not just the numbers.”	“Learned how to properly voice patient situation and report to professionals from a different discipline.”
Improvements for future	“There is still some confusion with calculating pharmacokinetic parameters differently for different drugs, and determining length of therapy for IV infusion/intermittent medications.”	“I can improve in my confidence concerning discussing patient findings with pharmacy. I want to always have it all together and there were some questions that my team did not have the answers to and that put down my confidence slightly.”
	“SBAR”	“SBAR”
	“I feel that I could better improve my communication skills and communicate in a more effective manner.”	“I feel like a little more planning on how to optimize the use of the nurses in the simulation would be very beneficial to the simulation experience for both professions.”
	“Truly being engaged when interacting with the nursing students and members of other health professions. Not feeling uncomfortable with things I am unfamiliar with.”	
	“Thinking on my feet.”	
	“Make sure that the nursing students know that what they are doing matters. Give them a bigger role in the simulation”	

**Table A2:** Students’ comparisons of simulation and lecture.

	Pharmacy	Nursing
What is the one thing you like better about lecture sessions as compared to simulation sessions?	“Learning the information to be used in application sessions.”	“It gives us the appropriate information to perform well in simulation.”
	“Lecture sessions set the foundation for us to understand the material.”	“You are given more detail and time to process information.”
	“I like actually having the information directly in front of me to be able to learn from.”	“Information is directly provided to us instead of us having to figure it out.”
		“Lecture sessions occur at a rate in which I expect.”
		“There are less expectations during lecture so I can focus 100% on the information being taught.”
What is the one thing you like better about simulation sessions as compared to lecture sessions?	“I like how it is hands-on application and gives me the ability to work with other people.”	“I learn well with hands on activities so simulations help me apply what I’ve read or learned in class to what I will actually be doing.”
	“Simulation give insight on real world situations that I see in work quite often. It is beneficial to see that we are being prepared based on real cases.”	“I like hands on learning and doing things physically helps me feel more comfortable in hospital situations because it gives me more practice.”
	“I enjoy a change of scenery and being able to improve my skills in different settings.”	“I love being hands-on. It is also easier to define my role as a nurse and the role of the pharmacist.”
		“It’s incredibly hands in and feels like you’re working with a real patient.”
